# Robotic Radiosurgery for Persistent Postoperative Acromegaly in Patients with Cavernous Sinus-Invading Pituitary Adenomas—A Multicenter Experience

**DOI:** 10.3390/cancers13030537

**Published:** 2021-01-31

**Authors:** Felix Ehret, Markus Kufeld, Christoph Fürweger, Alfred Haidenberger, Paul Windisch, Susanne Fichte, Ralph Lehrke, Carolin Senger, David Kaul, Daniel Rueß, Maximilian Ruge, Christian Schichor, Jörg-Christian Tonn, Günter Stalla, Alexander Muacevic

**Affiliations:** 1 Charité-Universitätsmedizin Berlin, Corporate Member of Freie Universität Berlin, Humboldt-Universität zu Berlin, and Berlin Institute of Health, Department of Radiation Oncology, 13353 Berlin, Germany; carolin.senger@charite.de (C.S.); david.kaul@charite.de (D.K.); 2 European Cyberknife Center, 81377 Munich, Germany; markus.kufeld@cyber-knife.net (M.K.); christoph.fuerweger@cyber-knife.net (C.F.); alfred.haidenberger@cyber-knife.net (A.H.); paul.windisch@ksw.ch (P.W.); alexander.muacevic@cyber-knife.net (A.M.); 3 Department of Radiation Oncology, Kantonsspital Winterthur, 8400 Winterthur, Switzerland; 4 CyberKnife Center Mitteldeutschland, 99089 Erfurt, Germany; susanne.fichte@ckcm.de; 5 German CyberKnife Center, 59494 Soest, Germany; RLehrke@barbaraklinik.de; 6 Charité-Universitätsmedizin Berlin, Corporate Member of Freie Universität Berlin, Humboldt-Universität zu Berlin, and Berlin Institute of Health, Charité CyberKnife Center, 13353 Berlin, Germany; 7 German Cancer Consortium (DKTK), Partner Site Berlin, German Cancer Research Center (DKFZ), 69120 Heidelberg, Germany; 8 Department of Stereotaxy and Functional Neurosurgery, Center for Neurosurgery, University Hospital Cologne, 50937 Cologne, Germany; daniel.ruess@uk-koeln.de (D.R.); maximilian.ruge@uk-koeln.de (M.R.); 9 Department of Neurosurgery, Ludwig-Maximilians-University Munich, 81377 Munich, Germany; christian.schichor@med.uni-muenchen.de (C.S.); joerg.christian.tonn@med.uni-muenchen.de (J.-C.T.); 10 Medicover Neuroendocrinology, 81667 Munich, Germany; guenter.stalla@medicover.de; 11 Department of Medicine IV, Ludwig-Maximilians-University Munich, 81377 Munich, Germany

**Keywords:** acromegaly, pituitary adenoma, radiosurgery, robotic radiosurgery, CyberKnife, growth hormone, insulin-like growth factor 1

## Abstract

**Simple Summary:**

Growth hormone-secreting tumors of the pituitary gland which infiltrate surrounding tissue structures may not be fully resectable. This causes many patients to suffer from acromegaly after an unsuccessful surgery. To limit the considerable morbidity and mortality of such patients, effective and safe treatment options are needed. Fractionated radiotherapy and growth hormone-lowering medication are possible treatment options. Robotic radiosurgery (RRS) may be a suitable treatment modality as well. However, only sparse and heterogeneous data are available. This first retrospective multicenter study investigated the efficacy and safety of RRS for this patient group. Outcomes provide evidence that RRS may achieve biochemical disease control or remission in most of the patients. The hormone levels are decreasing after treatment, whereas favorable risk and safety profiles of RRS were shown. No new tumor growth was observed throughout the available follow-up. These findings may guide future care for this challenging patient population.

**Abstract:**

Background: The rates of incomplete surgical resection for pituitary macroadenomas with cavernous sinus invasion are high. In growth hormone-producing adenomas, there is a considerable risk for persistent acromegaly. Thus, effective treatment options are needed to limit patient morbidity and mortality. This multicenter study assesses the efficacy and safety of robotic radiosurgery (RRS) for patients with cavernous sinus-invading adenomas with persistent acromegaly. Methods: Patients who underwent RRS with CyberKnife for postoperative acromegaly were eligible. Results: Fifty patients were included. At a median follow-up of 57 months, the local control was 100%. The pretreatment insulin-like growth factor 1 (IGF-1) levels and indexes were 381 ng/mL and 1.49, respectively. The median dose and prescription isodose were 18 Gy and 70%, respectively. Six months after RRS, and at the last follow-up, the IGF-1 levels and indexes were 277 ng/mL and 1.14, as well as 196 ng/mL and 0.83, respectively (*p* = 0.0001 and *p* = 0.0002). The IGF-1 index was a predictor for biochemical remission (*p* = 0.04). Nine patients achieved biochemical remission and 24 patients showed biochemical disease control. Three patients developed a new hypopituitarism. Conclusions: RRS is an effective treatment for this challenging patient population. IGF-1 levels are decreasing after treatment and most patients experience biochemical disease control or remission.

## 1. Introduction

With an incidence between 0.2 and 1.1 per 100,000 and year, acromegaly is a rare endocrinological disorder mostly caused by growth hormone (GH)-secreting adenomas of the pituitary gland, which lead to elevated insulin-like growth factor 1 (IGF-1) levels [1,2]. Approximately 75% of these endocrinologically active adenomas belong to the group of macroadenomas, which have a diameter of 10 mm or more [2]. As up to 70% of macroadenoma are considered to be infiltrating surrounding tissue and anatomical structures, complete surgical resection might not be feasible in approximately 20% of cases [3,4]. If gross surgical resection, the mainstay of treatment for GH-secreting macroadenomas, is not achievable, the risk of persisting hormone secretion and active disease remains. About 20% to 40% of patients may not achieve biochemical remission after surgery [5,6]. In patients with large macroadenomas and extensive invasion of surrounding anatomical structures, the risk is even higher given the lower chances of gross-total resection [5,7]. In patients with persistent postoperative acromegaly, adjuvant therapy options are needed and may include continuous and often lifelong medical therapy with IGF-1-lowering drugs as well as radiotherapy such as fractionated radiotherapy or radiosurgery [5,8]. Notably, medical treatment is cost-intensive and may not achieve disease control, while adverse events like hepatotoxicity, cholestatic, and digestive dysfunctions as well as skin rashes are commonly observed throughout the course of medication and in the setting of multidrug treatments [5,9]. Various studies have shown GammaKnife (GK)-based and conventional linear accelerator (LINAC)-based radiosurgery or hypofractionated radiosurgery to be potential treatment options besides fractionated radiotherapy for acromegaly [5,10,11,12,13,14,15]. Yet, only limited and heterogeneous data in regard to sample sizes, fractionation schemes and patient selection are available on the use of robotic radiosurgery (RRS) [12,13,16,17]. To the best of our knowledge, no dedicated radiosurgical multicenter study was conducted for this challenging patient subpopulation with cavernous sinus-invading pituitary adenomas so far. The objective of this retrospective study was to report the treatment and endocrinological outcomes for patients undergoing single-fraction RRS for persistent postoperative acromegaly caused by cavernous sinus-invading adenomas. Moreover, treatment-associated parameters as well as adverse events were assessed and analyzed.

## 2. Results

### 2.1. Patient Characteristics and Treatment Parameters

The median age at RRS was 47.6 years, ranging from 26 to 70 years. The majority of patients were male (56%). The median follow-up was 57.7 months, ranging from 6.1 to 171.9 months, with 22 patients (44%) having a follow-up of more than five years. Remaining residual tumor masses or presumed areas of vital tumor remnants were treated with a median dose of 18 Gray (Gy) prescribed to a median isodose of 70%, transforming into a median BED of 99 Gy and EQD2 of 66 Gy. The median irradiated volume was 1.38 cubic centimeters (cc). A median coverage of 98.4% was reached, whereas the conformity and homogeneity indexes were 1.3 and 1.4, respectively. The median maximum doses to the brainstem, optic nerve, and chiasm were all below 6 Gy. Five patients (10%) showed a tumor progression on MRI before RRS. The rest of patients (90%) showed a stable tumor prior to irradiation. Patient and treatment characteristics are summarized in Table 1 and Table 2.

### 2.2. Endocrinological Baseline

Before treatment delivery, 46 patients (92%) received medical treatment with various combinations of somatostatin-receptor ligands (SRLs), GH-receptor antagonists (PEG) or dopamine agonists (DA). Six and 16 patients showed visual deficits and various degrees of hypopituitarism, respectively. The most common pretreatment hormone deficits were related to gonadotropins (11 patients), the adrenocorticotropic hormone (ACTH) (nine patients), and the thyroid-stimulating hormone (TSH) (seven patients). One patient had a persistent antidiuretic hormone (ADH) deficiency after incomplete surgical tumor resection. The median baseline IGF-1 and IGF-1i levels were 381 ng/mL and 1.49, respectively. Thirteen patients (26%) had a higher IGF-1i than 2.25. At baseline, 41 patients (82%) had an uncontrolled disease. Nine patients (18%) showed a biochemically controlled disease as defined above but were suffering from active clinical disease and adverse events due to their continuous medical treatment. Subsequently, the indication for further treatment was confirmed. The majority of patients (92%) continued medication while receiving irradiation.

### 2.3. Treatment Outcome 

**Figure 1:**
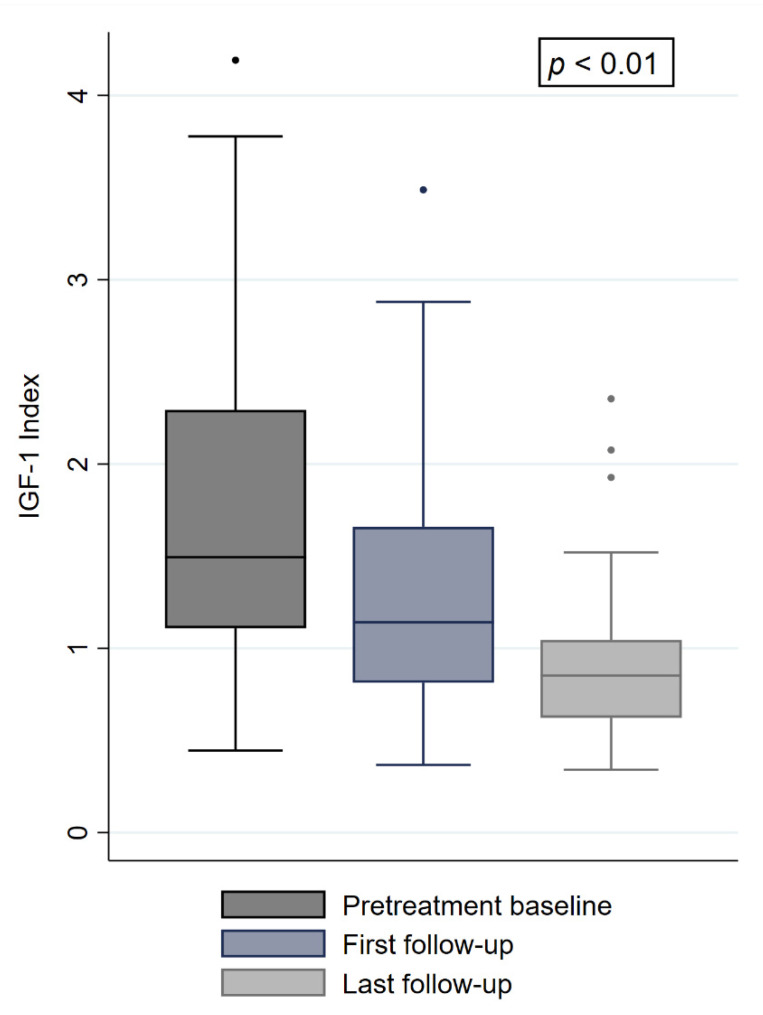
IGF-1i at baseline, first and last follow-up. “•” refers to outliers.

The local control (LC) throughout the follow-up was 100%, with nine patients (18%) showing a complete tumor regression at last follow-up. At the first follow-up, 6 months after irradiation, the median IGF-1 levels and the IGF-1i decreased by 27% and 23%, respectively (277 ng/mL and 1.14, *p* = 0.0001) (Figure 1). At the last available follow-up, both variables decreased further to a median IGF-1 level of 196 ng/mL and a median IGF-1i of 0.83 (−29% and −27%, *p* = 0.0002) (Figure 1). Of the initial 41 patients with an uncontrolled disease, 22 (53%) were biochemically controlled and five (12%) achieved biochemical remission. The remaining 14 patients had a persistent uncontrolled disease. Of the initial nine patients who had a biochemically controlled disease, four (44%) had a biochemical remission and two (22%) remained biochemically controlled. The remaining three patients (33%) showed mildly elevated IGF-1i of 1.02, 1.09, and 1.10 under continuous medical treatment and were classified as uncontrolled cases. A comparison of baseline characteristics of the four patients who had biochemical disease control before radiosurgery and showed biochemical remission after treatment only revealed a longer follow-up period in patients with remission (*p* = 0.01). In total, nine patients (18%) showed biochemical remission, 24 (48%) were biochemically controlled, and 17 (34%) uncontrolled at last follow-up, with 38 patients (76%) taking SRLs, PEG, or DA. Notably, nine (53%) of the uncontrolled patients had an IGF-1i of less than 1.2 and only four (23%) an index of more than 1.5. All patients with biochemical remission had a pretreatment IGF-1i of less than 2.25. Throughout the follow-up, three patients (6%) developed a new onset of hypopituitarism. In total, new hormone deficits were related to gonadotropins (two patients), ACTH (two patients), TSH (three patients), and ADH (one patient). No worsening of a preexisting hypopituitarism was observed. No patient suffering from a pretreatment hormone insufficiency recovered, leading to a total of 19 patients (38%) with hypopituitarism at the last follow-up. Two patients with pretreatment visual deficits recovered after irradiation due to tumor shrinkage in the cavernous sinus. One patient (2%) received a second single-fraction irradiation after 18 months due to exacerbation of symptoms and active disease, with no visible tumor growth or progression on imaging. This patient had an additional follow-up of 40 months after the second treatment and achieved a biochemical controlled disease. Baseline comparisons between patients with and without biochemical remission at last follow-up revealed differences in pretreatment IGF-1 levels and IGF-1i (*p* = 0.03 and *p* = 0.02, Table 3). In the univariable and multivariable analysis to predict biochemical remission at last follow-up, the pretreatment IGF-1i showed statistical significance (*p* = 0.04) (Table 4). Sex showed a trend towards significance (*p* = 0.058), with seven of the nine patients (77%) with biochemical remission being female. The remaining variables, including age, irradiated volume, dose, maximum dose, minimum dose, respective BED, and complete tumor regression had no significant impact (Table 4). Besides the events mentioned above, no acute or late toxicity was observed.

## 3. Discussion

As previously reported and discussed, radiosurgical results for acromegaly can greatly vary [8,10]. With the vast majority of reported studies investigating GK- and conventional LINAC-based radiosurgery, RRS and its outcomes have been scarcely described. Herein, we report our multicenter experience for a relatively rare subpopulation of patients exclusively suffering from cavernous sinus-invading adenomas with persistent postoperative acromegaly after RRS. In regard to the outcomes after radiosurgery, a recent retrospective multicenter trial of the International Gamma Knife Research Foundation with 371 patients showed sound results as IGF-1 lowering medication was held in 56% of patients who were on medical treatment before GK-based radiosurgery, with 59% of patients demonstrating durable endocrine remission [10]. Comparable results were observed in a single-center trial from the Mayo Clinic, with 57% of treated patients achieving biochemical remission without the need for further medication [11]. Kim et al. reported on the results of GK-based radiosurgery for cavernous sinus-invading pituitary adenomas. In the study cohort of 30 patients, 14 (46%) achieved biochemical remission, with a considerable number of patients developing a new hypopituitarism after treatment [15]. Previous RRS studies on acromegaly have shown remission rates between 17 and 59% [12,13,16,17]. Notably, the majority of these and other previous studies included patients who did not undergo primary surgical resection or had non-invading micro- as well as macroadenomas. Moreover, the RRS studies also included patients receiving varying fractionation schemes, had different tumor sizes and used differing definitions for disease control and biochemical remission, highlighting the general issue of data heterogeneity in acromegaly studies. 

In this study, 18% and 48% of patients achieved biochemical remission and were biochemically controlled, respectively. These rates are lower compared to the previously mentioned reports. Considering our patient selection and treatment procedure, three factors may have influenced these outcomes. First, this study only included patients with cavernous sinus-invading tumor remnants of previous macroadenomas after unsuccessful total surgical resection. Most of them required further medical treatment. This indicates a selection of a rather challenging and unfavorable patient cohort as less than 20% of these patients will be cured by surgery alone [13,18,19,20,21]. Second, there is an ongoing debate about pausing IGF-1 lowering medication during treatment delivery due to worse outcomes, initially described by Landolt et al. in 2000 [22,23]. Since then, varying results have been reported, whereas recent radiosurgical studies recommend cessation of medication up to eight weeks before treatment if no medical contraindication is apparent [10,11,24,25]. Notably, guidelines by the Endocrine Society and the American Association of Clinical Endocrinologists have not included this recommendation [26]. Subsequently, and in regard to some of our patients’ clinical status, medical therapy was not stopped in 92% of cases. In contrast, past studies have had considerable proportions of patients stopping their medication before irradiation and identified temporary cessation as an independent factor of initial and durable biochemical remission [10,13]. At this point, we cannot provide firm conclusions on this matter but suggest evaluating this subject prospectively if medically feasible and appropriate. Third, a higher BED was found in patients with biochemical remission compared to uncontrolled patients (mean 163 Gy vs. 111 Gy, α/β ratio = 4) [13]. Herein, the median and mean BED were 99 Gy and 104 Gy, respectively, and no significant differences were found between patients with and without biochemical remission. This may be explained by the rather homogeneous dose prescriptions in our study cohort, potentially masquerading a subset of patients that would profit from a higher BED. A recent study found BED to better correlate with the endocrinological outcome than the prescribed dose alone [11]. Eventually, these circumstances and factors may have affected our endocrinological outcomes and the total number of patients with biochemical remission. 

Nevertheless, post-treatment IGF-1 levels and IGF-1i showed a significant decrease over time, as previously reported by other studies. However, the potentially delayed treatment effect should be kept in mind, especially when counseling affected patients. Lowering the IGF-1 levels is a crucial objective in the management of patients with persistent acromegaly, as higher values are linked with higher mortality [27]. In general, the IGF-1 levels, even when adjusted to the age and sex of the patient, may lead to systemic biases given changes in testing over time due to the use of different assays and kit manufacturers. Thus, the use of the IGF-1i was recommended to account for the variability in IGF-1 testing and to improve interpretation of results among different studies [11]. The index was initially reported by Gutt et al. [28]. So far, a limited number of radiosurgical studies have used the index to depict their findings. However, in all the five studies reporting the IGF-1i, it was found to be a reliable predictor of biochemical remission [11,25,29,30,31]. This finding was also reproduced in our study as the index was found to significantly predict biochemical remission of patients at last follow-up, with patients showing a lower index having higher chances of remission. Thus, we agree with the conclusion of Graffeo et al. and suggest including the IGF-1i in future, ideally prospective, studies on acromegaly [11]. Moreover, we recommend implementing frequent GH measurements to complement the IGF-1 levels as indicated by the Acromegaly Consensus Conference to provide more insights on their relationship after radiotherapy or radiosurgery [32]. 

Finally, and to the best of our knowledge, this is the first radiosurgical multicenter study explicitly dedicated to intracavernous adenomas after surgical resection with persisting acromegaly. Given previous studies on the efficacy of radiosurgery for acromegaly in general, its role for this challenging patient cohort has not been extensively analyzed to date [15]. This is mainly caused by the rarity of such patients and by the data heterogeneity of previous reports. Moreover, and in contrast to the majority of previous radiosurgical studies on this topic, RRS was utilized, not GK- or conventional LINAC-based radiosurgery. As described by Sala et al., it is not certainly known if GK-based results can be extrapolated to RRS. Herein, we provided a first retrospective multicenter experience with RRS and observed promising results. Particular strengths of this study comprise the homogeneity of included patients, the sample size, the follow-up duration, and the treatment procedure itself, which was limited to just one fraction for each treatment. In contrast, previous studies utilizing RRS partly included patients that were primarily and secondarily treated with radiosurgery [13]. Moreover, not all studies reported on the extension and possible infiltration of the treated tumors [12,16,17]. Finally, different and varying fractionation schemes have been commonly applied in previous works [12,13,16,17]. Despite these strengths, several limitations and potential sources of confounding and sampling biases of this study may be apparent given the changes in IGF-1 assessments over time and its retrospective study design. Despite the recommendation of including GH into respective analyses, we cannot provide comprehensive GH measurements for our study cohort after RRS. This is mainly due to infrequent assessments throughout the follow-up of patients. All these factors may limit the drawn conclusions of this study.

## 4. Materials and Methods 

### 4.1. Patients

Between June 2005 and June 2020, 50 patients from five departments were eligible for analysis in this retrospective multicenter study. All patients underwent the attempt of gross surgical resection for a GH-secreting macroadenoma cavernous sinus invasion causing elevated IGF-1 levels. An elevated IGF-1 level was defined as an IGF-1 value above the age- and sex-adjusted upper normal limit. Macroadenomas were defined as pituitary gland adenomas with a diameter of at least 10 mm in thin-sliced magnetic resonance imaging (MRI) before surgery. Invasion of the cavernous sinus was confirmed with the help of imaging data (MRI/computed tomography (CT)) and operation reports. After surgery, all patients had persisting acromegaly, diagnosed by a board-certified endocrinologist and in accordance with the respective diagnosis guidelines at the time of diagnosis. Subsequently, the indication for radiosurgery was confirmed in an interdisciplinary tumor board consisting of neuroradiologists, neurosurgeons, neuropathologists, and radiation oncologists. Patient information, including medical history, lab, and follow-up data, was stored at each center in the respective electronic health records or patient files. Only patients with a follow-up of at least six months and who did not receive any prior radiotherapy throughout their course of disease were included in the analysis.

### 4.2. Endocrinological Analysis

Given the proven reliability of IGF-1 as a diagnostic and monitoring marker for acromegaly, it was used as the primary biomarker in this study to analyze endocrinological outcomes [32,33,34,35]. IGF-1 values were adjusted for age and sex and subsequently transformed into the IGF-1 index (IGF-1i) by dividing each adjusted serum IGF-1 value by the upper limit of the reference range for IGF-1 [11,25,30,31,36]. IGF-1 analyses included values prior to RRS, during the first follow-up six months after treatment delivery and at the last available follow-up. Biochemically controlled disease was defined as IGF-1 levels within normal limits with medication, whereas biochemical remission was defined as IGF-1 levels within normal limits without the need for further medication. As previous studies indicated that an IGF-1i higher than 2.25 is associated with a worse endocrinological outcome, the number of respective patients was also calculated herein [11,25]. After treatment delivery, the onset of a new hypopituitarism was assessed by measuring the respective target hormone levels, with the respective diagnosis made by a board-certified endocrinologist. The necessity of a new or intensified hormone replacement therapy at the last available follow-up was defined as a new treatment-associated hypopituitarism.

### 4.3. Treatment Procedure and Outcome

Prior to RRS, patients underwent a planning CT scan of the head with 1 mm slice thickness and contrast agent. The CT was subsequently fused with secondary MRI, including contrast-enhanced T1 sequences, with 1 mm slice thickness. Treatment planning was performed with various versions of the MultiPlan and Precision software (Accuray Inc., Sunnyvale, CA, USA). All treatments were delivered using a CyberKnife^®^ robotic radiosurgery system (Accuray Inc., Sunnyvale, CA, USA). All patients underwent RRS with one fraction. Treatment parameters including coverage; conformity index; heterogeneity index; minimum, mean, and maximum tumor doses were extracted for analyses. LC was defined as the absence of any tumor growth on MRI during follow-up scans. Complete tumor regression was defined as the absence of visible tumor volume on the last available contrast-enhanced MRI follow-up. Biologically effective dose (BED) and the 2 gray (Gy) equivalent dose (EQD2) were calculated according to Fowler, with an α/β ratio of 4, as the pituitary adenoma cells were classified as late responding tissue [37,38].

### 4.4. Statistical Analysis

Continuous variables were reported as means with standard deviation (SD), and categorical variables were reported as frequencies with their respective percentage. The data were tested for normal distribution by visual appearance, skewness, kurtosis, and the Shapiro–Wilk test. Accordingly, two-sided paired und unpaired student’s t-tests, Wilcoxon signed-rank tests, Wilcoxon rank-sum tests, Kruskal–Wallis tests, and logistic regressions analyses were performed. As this study is aiming towards the generation of hypotheses rather than hypothesis confirmation, statistical tests and results were not adjusted and corrected for multiple testing. All tests were conducted using STATA 16.0 MP (StataCorp, College Station, TX, USA). Statistical significance was set at a *p*-value equal or less than 0.05.

## 5. Conclusions

Single-fraction RRS is a safe and effective treatment modality for this challenging endocrinological patient cohort. An excellent LC was observed, whereas the IGF-1 levels and IGF-1i significantly decreased after treatment delivery. Biochemical control or remission of acromegaly was seen in 66% of patients, while the treatment showed a favorable safety profile. Patients with a high IGF-1i before RRS may have limited chances of biochemical remission or biochemical disease control.

## Figures and Tables

**Table 1:** Patient characteristics.

Total Number of Patients	50		
Sex (Male/Female, %)	28 (56)		22 (44)
	Mean (±SD)	Median	Range
Age (years)	46.7 (10.3)	47.6	26.8–70.7
Pretreatment Karnofsky Performance Status (%)	93.3 (8.5)	90	60–100
Follow-up (months)	57.3 (42.4)	57.7	6.1–171.9
IGF-1 level before RRS (ng/mL)	439 (238)	381	98–1161
IGF-1i before RRS	1.73 (0.90)	1.49	0.44–4.19
IGF-1 level at 6-month follow-up (ng/mL)	322 (166)	277	109–858
IGF-1i at 6-month follow-up	1.27 (0.64)	1.14	0.36–3.48
IGF-1 level at last follow-up (ng/mL)	226 (99)	196	89–575
IGF-1i at last follow-up	0.89 (0.40)	0.83	0.34–2.35
Patients with an IGF-1i larger 2.25 before RRS (%)	13 (26)		
Patients with medication before RRS (%)	46 (92)		
Patients with medication during RRS (%)	46 (92)		
Patients with medication at last follow-up (%)	38 (76)		
Pretreatment visual changes (%)	6 (12)		
Post-treatment visual changes (%)	4 (8)		
Pretreatment hypopituitarism (%)	16 (32)		
Post-treatment hypopituitarism (%)	19 (38)		
Patients with biochemically controlled disease before RRS	9 (18)		
Patients with biochemically controlled disease at last follow-up (%)	24 (48)		
Patients with biochemical remission at last follow-up (%)	9 (18)		

**Table 2:** Treatment characteristics.

Variable	Median	Mean	Range
Irradiated/tumor volume (cc)	1.38	2.07	0.13–12.00
Prescription dose (Gy)	18	18.4	14–24
Prescription isodose (%)	70	69.7	52–80
Max tumor dose (Gy)	25.7	26.8	21.4–43.6
Min tumor dose (Gy)	15.3	15.3	7.0–24.1
Mean tumor dose (Gy)	21.6	22.1	17.1–31.2
Conformity index	1.3	1.3	1.0–2.2
Homogeneity index	1.4	1.4	1.2–1.9
Coverage (%)	98.4	96.2	83.0–100.0
Max optic nerve dose (Gy)	5.8	6.0	1.1–15.5
Max chiasm dose (Gy)	5.7	5.7	1.1–15.5
Max brainstem dose (Gy)	5.1	5.7	0.0–17.6
BED (Gy)	99.0	104.5	63.0–168.0
EQD2 (Gy)	66.0	69.6	42.0–112.0

**Table 3:** Baseline comparison between patients with and without biochemical remission (uncontrolled and biochemically controlled patients).

Variable	Biochemical Remission (±SD)	Without Biochemical Remission (±SD)	*p*-Value
Age	47.6 (12.5)	46.5 (9.9)	0.77
IGF-1 levels before treatment (ng/mL)	290.2 (138.3)	472.6 (243.8)	0.03
IGF-1i before treatment	1.11 (0.4)	1.86 (0.9)	0.02
Irradiated/tumor volume (cc)	1.2 (0.6)	2.2 (2.5)	0.24
Prescription dose (Gy)	18.7 (3.1)	18.3 (1.5)	0.58
Prescription isodose (%)	70.5 (7.6)	69.5 (6.8)	0.70
Max dose in tumor (Gy)	26.9 (6.9)	26.8 (4.0)	0.95
Min dose in tumor (Gy)	14.8 (4.6)	15.4 (3.5)	0.65
Mean dose in tumor (Gy)	22.3 (4.3)	22.0 (2.6)	0.90
Coverage (%)	94.7 (5.5)	96.5 (4.1)	0.26
BED (Gy)	109.0 (33.5)	103.5 (16.7)	0.46

**Table 4:** Logistic regression analyses for patients with biochemical remission at last follow-up.

Biochemical Remission (Univariable Analysis)			
Factor	Odds Ratio	*p*-Value	95% Confidence Interval
Age	1.01	0.76	0.94–1.08
Sex	0.16	0.03	0.03–0.89
Irradiated/tumor volume (cc)	0.64	0.26	0.29–1.39
Dose (Gy)	1.10	0.57	0.76–1.59
Max dose in tumor (Gy)	1.00	0.95	0.86–1.17
Mean dose in tumor (Gy)	1.03	0.75	0.81–1.31
Min dose in tumor (Gy)	0.95	0.64	0.78–1.16
BED (Gy)	1.01	0.46	0.97–1.04
Pretreatment IGF Index	0.15	0.03	0.02–0.91
Pretreatment IGF level (ng/mL)	0.99	0.05	0.98–1.00
IGF-1i at 1. follow-up	0.17	0.06	0.02–1.10
IGF-1 level at 1. follow-up (ng/mL)	0.99	0.08	0.98–1.00
Complete tumor regression	2.91	0.19	0.56–14.94
**Biochemical Remission (Multivariable Analysis)**			
Age	0.84	0.13	0.67–1.05
Sex	0.01	0.05	0.01–1.23
Irradiated/tumor volume (cc)	0.07	0.11	0.01–1.91
Dose (Gy)	2.35	0.45	0.25–21.80
Max dose in tumor (Gy)	1.11	0.75	0.25–2.06
Mean dose in tumor (Gy)	(co-linear with dose)		
Min dose in tumor (Gy)	0.64	0.22	0.32–1.30
BED (Gy)	(co-linear with dose)		
Pretreatment IGF-1i	0.04	0.04	0.01–0.93
Pretreatment IGF-1 level (ng/mL)	(co-linear with pretreatment IGF Index)		
IGF-1i at 1. follow-up	(co-linear with pretreatment IGF Index)		
IGF-1 level at 1. follow-up (ng/mL)	(co-linear with pretreatment IGF Index)		
Complete tumor regression	8.06	0.27	0.19–39.2

## Data Availability

The data that support the findings of this study are available from the corresponding author, F.E., upon reasonable request.

## References

[1] Lavrentaki A., Paluzzi A., Wass J.A., Karavitaki N. (2017). Epidemiology of acromegaly: Review of population studies. Pituitary.

[2] Molitch M.E. (2017). Diagnosis and Treatment of Pituitary Adenomas: A Review. Jama.

[3] Nomikos P., Buchfelder M., Fahlbusch R. (2005). The outcome of surgery in 668 patients with acromegaly using current criteria of biochemical ‘cure’. Eur. J. Endocrinol..

[4] Meij B.P., Lopes M.B., Ellegala D.B., Alden T.D., Laws E.R. (2002). The long-term significance of microscopic dural invasion in 354 patients with pituitary adenomas treated with transsphenoidal surgery. J. Neurosurg..

[5] Mehta G.U., Lonser R.R. (2017). Management of hormone-secreting pituitary adenomas. Neuro-oncology.

[6] Starke R.M., Raper D.M., Payne S.C., Vance M.L., Oldfield E.H., Jane J.A. (2013). Endoscopic vs microsurgical transsphenoidal surgery for acromegaly: Outcomes in a concurrent series of patients using modern criteria for remission. J. Clin. Endocrinol Metab.

[7] Juraschka K., Khan O.H., Godoy B.L., Monsalves E., Kilian A., Krischek B., Ghare A., Vescan A., Gentili F., Zadeh G. (2014). Endoscopic endonasal transsphenoidal approach to large and giant pituitary adenomas: Institutional experience and predictors of extent of resection. J. Neurosurg..

[8] Ding D., Starke R.M., Sheehan J.P. (2014). Treatment paradigms for pituitary adenomas: Defining the roles of radiosurgery and radiation therapy. J. Neurooncol..

[9] Tritos N.A., Biller B.M.K. (2017). Pegvisomant: A growth hormone receptor antagonist used in the treatment of acromegaly. Pituitary.

[10] Ding D., Mehta G.U., Patibandla M.R., Lee C.C., Liscak R., Kano H., Pai F.Y., Kosak M., Sisterson N.D., Martinez-Alvarez R. (2019). Stereotactic Radiosurgery for Acromegaly: An International Multicenter Retrospective Cohort Study. Neurosurgery.

[11] Graffeo C.S., Donegan D., Erickson D., Brown P.D., Perry A., Link M.J., Young W.F., Pollock B.E. (2020). The Impact of Insulin-Like Growth Factor Index and Biologically Effective Dose on Outcomes After Stereotactic Radiosurgery for Acromegaly: Cohort Study. Neurosurgery.

[12] Iwata H., Sato K., Nomura R., Tabei Y., Suzuki I., Yokota N., Inoue M., Ohta S., Yamada S., Shibamoto Y. (2016). Long-term results of hypofractionated stereotactic radiotherapy with CyberKnife for growth hormone-secreting pituitary adenoma: Evaluation by the Cortina consensus. J. Neurooncol..

[13] Sala E., Moore J.M., Amorin A., Martinez H., Bhowmik A.C., Lamsam L., Chang S., Soltys S.G., Katznelson L., Harsh G.R. (2018). CyberKnife robotic radiosurgery in the multimodal management of acromegaly patients with invasive macroadenoma: A single center’s experience. J. Neurooncol..

[14] Rhome R., Germano I.M., Sheu R.D., Green S. (2017). Long-term outcomes of acromegaly treated with fractionated stereotactic radiation: Case series and literature review. Neurooncol. Pract..

[15] Kim E.H., Oh M.C., Chang J.H., Moon J.H., Ku C.R., Chang W.S., Lee E.J., Kim S.H. (2018). Postoperative gamma knife radiosurgery for cavernous sinus-invading growth hormone-secreting pituitary adenomas. World Neurosurg..

[16] Roberts B.K., Ouyang D.L., Lad S.P., Chang S.D., Harsh G.R.t., Adler J.R., Soltys S.G., Gibbs I.C., Remedios L., Katznelson L. (2007). Efficacy and safety of CyberKnife radiosurgery for acromegaly. Pituitary.

[17] Cho C.B., Park H.K., Joo W.I., Chough C.K., Lee K.J., Rha H.K. (2009). Stereotactic Radiosurgery with the CyberKnife for Pituitary Adenomas. J. Korean Neurosurg. Soc..

[18] Leach P., Abou-Zeid A.H., Kearney T., Davis J., Trainer P.J., Gnanalingham K.K. (2010). Endoscopic transsphenoidal pituitary surgery: Evidence of an operative learning curve. Neurosurgery.

[19] Dusek T., Kastelan D., Melada A., Baretic M., Skoric Polovina T., Perkovic Z., Giljevic Z., Jelcic J., Paladino J., Aganovic I. (2011). Clinical features and therapeutic outcomes of patients with acromegaly: Single-center experience. J. Endocrinol. Investig..

[20] Rieger A., Rainov N.G., Ebel H., Sanchin L., Shibib K., Helfrich C., Hoffmann O., Burkert W. (1997). Factors predicting pituitary adenoma invasiveness in acromegalic patients. Neurosurg. Rev..

[21] Wang M., Mou C., Jiang M., Han L., Fan S., Huan C., Qu X., Han T., Qu Y., Xu G. (2012). The characteristics of acromegalic patients with hyperprolactinemia and the differences in patients with merely GH-secreting adenomas: Clinical analysis of 279 cases. Eur. J. Endocrinol..

[22] Landolt A.M., Haller D., Lomax N., Scheib S., Schubiger O., Siegfried J., Wellis G. (1998). Stereotactic radiosurgery for recurrent surgically treated acromegaly: Comparison with fractionated radiotherapy. J. Neurosurg..

[23] Landolt A.M., Haller D., Lomax N., Scheib S., Schubiger O., Siegfried J., Wellis G. (2000). Octreotide may act as a radioprotective agent in acromegaly. J. Clin. Endocrinol Metab.

[24] Sheehan J.P., Pouratian N., Steiner L., Laws E.R., Vance M.L. (2011). Gamma Knife surgery for pituitary adenomas: Factors related to radiological and endocrine outcomes. J. Neurosurg.

[25] Pollock B.E., Jacob J.T., Brown P.D., Nippoldt T.B. (2007). Radiosurgery of growth hormone-producing pituitary adenomas: Factors associated with biochemical remission. J. Neurosurg..

[26] Katznelson L., Laws E.R., Melmed S., Molitch M.E., Murad M.H., Utz A., Wass J.A.H. (2014). Acromegaly: An Endocrine Society Clinical Practice Guideline. J. Clin. Endocrinol. Metab..

[27] Holdaway I.M., Bolland M.J., Gamble G.D. (2008). A meta-analysis of the effect of lowering serum levels of GH and IGF-I on mortality in acromegaly. Eur. J. Endocrinol..

[28] Gutt B., Wowra B., Alexandrov R., Uhl E., Schaaf L., Stalla G.K., Schopohl J. (2005). Gamma-knife surgery is effective in normalising plasma insulin-like growth factor I in patients with acromegaly. Exp. Clin. Endocrinol. Diabetes.

[29] Kong D.S., Kim Y.H., Kim Y.H., Hur K.Y., Kim J.H., Kim M.S., Paek S.H., Kwon D.H., Kim D.K., Lee J.I. (2019). Long-Term Efficacy and Tolerability of Gamma Knife Radiosurgery for Growth Hormone-Secreting Adenoma: A Retrospective Multicenter Study (MERGE-001). World Neurosurg.

[30] Liu X., Kano H., Kondziolka D., Park K.J., Iyer A., Niranjan A., Flickinger J.C., Lunsford L.D. (2012). Gamma knife radiosurgery for clinically persistent acromegaly. J. Neurooncol..

[31] Losa M., Gioia L., Picozzi P., Franzin A., Valle M., Giovanelli M., Mortini P. (2008). The role of stereotactic radiotherapy in patients with growth hormone-secreting pituitary adenoma. J. Clin. Endocrinol. Metab..

[32] Melmed S., Bronstein M.D., Chanson P., Klibanski A., Casanueva F.F., Wass J.A.H., Strasburger C.J., Luger A., Clemmons D.R., Giustina A. (2018). A Consensus Statement on acromegaly therapeutic outcomes. Nat. Rev. Endocrinol..

[33] Cook D., Yuen K., Biller B., Kemp S., Vance M. (2009). American Association of Clinical Endocrinologists Medical Guidelines for Clinical Practice for Growth Hormone Use in Growth Hormone-Deficient Adults and Transition Patients-2009 Update: Executive Summary of Recommendations. Endocr. Pract..

[34] Subbarayan S.K., Fleseriu M., Gordon M.B., Brzana J.A., Kennedy L., Faiman C., Hatipoglu B.A., Prayson R.A., Delashaw J.B., Weil R.J. (2012). Serum IGF-1 in the diagnosis of acromegaly and the profile of patients with elevated IGF-1 but normal glucose-suppressed growth hormone. Endocr. Pract..

[35] Brabant G. (2003). Insulin-like growth factor-I: Marker for diagnosis of acromegaly and monitoring the efficacy of treatment. Eur. J. Endocrinol..

[36] ElmLinger M.W., Kuhnel W., Weber M.M., Ranke M.B. (2004). Reference ranges for two automated chemiluminescent assays for serum insulin-like growth factor I (IGF-I) and IGF-binding protein 3 (IGFBP-3). Clin. Chem. Lab. Med..

[37] Fowler J.F. (1989). The linear-quadratic formula and progress in fractionated radiotherapy. Br. J. Radiol..

[38] Fowler J.F. (2010). 21 years of biologically effective dose. Br. J. Radiol..

